# Evaluating the various phases of cisplatin-induced emesis in rats

**DOI:** 10.3892/ol.2014.2506

**Published:** 2014-09-05

**Authors:** JUN SHI

**Affiliations:** Department of Traditional Chinese Medicine, Shanghai Changzheng Hospital, The Second Military Medical University, Shanghai 200003, P.R. China

**Keywords:** conditioned taste aversion, pica, anorexia, cisplatin-induced emesis, rat

## Abstract

Use of cisplatin as a chemotherapeutic agent causes acute and delayed emesis. Kaolin, saccharin solution and normal feed consumption have been evaluated as an index of cisplatin-induced emesis in rats; however, the most preferable of these methods for evaluating the various phases of emesis remains unclear. In the current study, kaolin, saccharin solution and normal feed consumption following cisplatin administration (6 mg/kg intraperitoneally) were simultaneously investigated in rats. Kaolin consumption increased significantly following cisplatin administration and was attenuated by granisetron administration 0–24 h following the injection. Saccharin solution consumption, however, decreased significantly 0–48 h following cisplatin administration, however, was attenuated by administration of granisetron within 0–24 h only. A reduced intake of normal feed was observed in the control group and was reversed by granisetron within the 0–72 h period. The present study indicates that kaolin consumption may be evaluated as an index of cisplatin-induced acute emesis and saccharin solution consumption may be evaluated as an index of delayed emesis, while normal feed consumption as an indicator of anorexia nervosa may be suitable to evaluate all phases of emesis and serve as an indicator of quality of life.

## Introduction

Cancer chemotherapy is frequently accompanied by severe emesis. Cisplatin is an emetic, platinum-based chemotherapeutic agent that has been widely used to treat various malignancies. Emetic agents, including cisplatin, have been shown to induce taste aversion, kaolin ingestion behavior (pica) and anorexic behavior in a diverse range of species (1–3). Rodents, such as rats, are incapable of vomiting, however, investigations have demonstrated that rats readily exhibit analogous behaviors in response to cisplatin administration. These behaviors were associated with multiple neuropharmacological mechanisms, including the dopamine D2 receptors in the chemoreceptor trigger zone, the 5-HT_3_ receptors in the visceral afferents of the stomach wall (4), the vagal afferents of the common hepatic branch (5), the midbrain and brainstem (6), the hippocampus, hypothalamus, and medulla oblongata (7) and the emetic system of the lateral parabrachial nucleus (8). Therefore, the predominant species for investigating emesis has been the laboratory rat (9–12).

Rats exhibit pica in response to stimuli, which ordinarily induce emesis in species that posess an emetic reflex (13), hence, kaolin consumption has been evaluated as an index of cisplatin-induced emesis in rats. It has also been demonstrated, using rotation stimulation, that pica and conditioned taste aversion (CTA) are behavioral changes, which are specific to rats (14). Previously, cisplatin was used as the standard emetic agent in studies of CTA learning in rats (15,16), with saccharin solution consumption, to indicate CTA, used as an alternative index of emesis in rats. Anorexia nervosa is one of the most unwanted side-effects in cancer patients who are undergoing chemotherapy. It was found that rats exhibited anorexic behavior in response to cisplatin administration (5,17); therefore, normal feed intake has been evaluated in the present study as a third index of cisplatin-induced emesis in rats.

Cisplatin administration causes acute and delayed nausea and vomiting via different mechanisms. Although the gastric retention of solid material (18), as well as radiological methods (19), have been reported as novel indicators for predicting the potential of compounds that induce emesis in non-vomiting rodents; pica, CTA and normal feed consumption are commonly used as markers in investigations of emesis. However, which of these markers is most suitable for assessing the availability of cisplatin-induced acute or delayed vomiting remains unclear. Thus, the aim of the current study was to investigate which index, of kaolin, saccharin solution or normal feed consumption, is the most suitable for evaluating the various phases of cisplatin-induced emesis.

## Materials and methods

### Animals

Sixty adult Wistar strain rats (equal numbers of males and females), weighing between 200 and 220 g, were purchased from The Shanghai Slac Laboratory Animal Company Ltd. (Shanghai, China) and acclimated to the laboratory environments for one week prior to the investigation. Rats were housed in a polycarbonate cage (45×35×25 cm), and maintained under controlled conditions of temperature (22±2°C), relative humidity (55±10%) and lighting (12-h light/dark cycle). All animals were allowed free access to kaolin, saccharin solution and standard rat chow pellets (Shanghai Slac Laboratory Animal Company Ltd.). The standard rat chow pellets, kaolin and saccharin were all placed in separate containers throughout the experiment to prevent them mixing and to enable the more accurate calculation of weight. All cages were fitted with a wire mesh floor to permit the collection of spilt kaolin and feed. This study was approved by the ethics committee of the Second Military Medical University (Shanghai, China).

### Materials

Kaolin was prepared using a standard protocol from the literature with slight modifications (13,20,21). Briefly, 50 g pharmacological grade kaolin (China Clay [naturally hydrated aluminum silicate]; Tianjin Third Chemical Reagent Factory, Tianjin, China) was mixed with 1 g of acacia (gum arabic; Guangfu Fine Chemical Research Institute, Tianjin, China) (50:1 ratio) with distilled water to form a thick paste. The paste was extruded through a syringe onto wire mesh trays to dry partially and was then introduced into a column, which was the same shape as the rat chow pellets. The kaolin pellets were placed on plastics trays and completely dried at room temperature for 72 h.

The saccharin (0.15% w/v) and granisetron solutions were premixed using deionized water. Reagent grade granular sodium saccharin anhydrous (Northern Food Co., Ltd., Tianjin, China) and granisetron hydrochloride dispersible tablets (1 mg/tablet; Guangxi Pubei Pharmaceutical Factory, Guangxi, China) were used.

### Procedures

Male and female rats were randomly divided into three groups; blank, control and granisetron, each consisting of 20 animals. Kaolin pellets and saccharin solution were placed on the stainless steel grid cover of the cage for six days (days 1–6) prior to cisplatin injection to allow the rats to adapt to its presence, and all animals received intragastric administration of physiological saline for three days (days 4–6; dosage, 2 ml/day), prior to administration of the cisplatin injection. Between days 7 and 9, the granisetron group received intragastric administration of the granisetron solution (2.7 mg/kg body weight; dosage, 2 ml/day), whilst the other two groups continued to receive physiological saline. On day 7, cisplatin (6 mg/kg via intraperitoneal [i.p.] injection) was administered to all rats, with the exception of the blank group, 1 h subsequent to the intragastric administration of granisetron. The kaolin, saccharin solution and normal feed containers were removed each day (at 9:00 am). The kaolin and normal feed was collected and weighed, and the weight of the bottle of saccharin was also determined. The quantity of kaolin, normal feed and saccharin solution consumed during each 24 h period was determined by comparing the weights with the initial weights. The following formula was used to determine the ultimate weight value: Ultimate weight value (day n) = weight (day n) − weight (day n+1). The total observation period was 72 h.

### Statistical analysis

Data are presented as the mean values ± standard deviation. Kaolin, saccharin solution and normal feed consumption were compared between the three groups using a two-way repeated measures analysis of variance followed by Fisher’s least significant difference multiple comparison tests. Data regarding the heterogeneity of variance or abnormal distribution were compared using a Kruskal-Wallis rank sum test followed by Nemenyi multiple comparison tests. P<0.05 was considered to indicate a statistically significant difference.

## Results

### Kaolin consumption in rats

**Figure 1:**
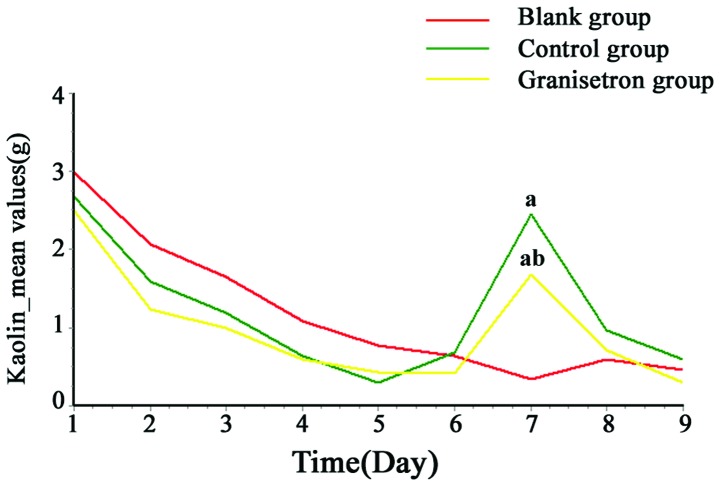
Effect of cisplatin administration on kaolin consumption in rats. Cisplatin significantly increased kaolin consumption by day 7 only, and this effect was attenuated by granisetron administration (day 7). ^a^P<0.01 vs. blank group; ^b^P<0.05 vs. control group.

On days 1 and 2, all of the rats showed abnormal pica behavior, however, kaolin consumption gradually returned to the control level (~0.3–0.7 g/day), which was recorded prior to the start of the experiment. No significant difference was identified among the three groups between days 1 and 6 (P>0.05). As demonstrated by Table I and Fig. 1, following the cisplatin injection, kaolin consumption in the control and granisetron groups increased significantly when compared with that of the blank group at 24 h (day 7; P<0.01), however not at 48 h (day 8; P=0.2658) or 72 h (day 9; P=0.1222). The increase in kaolin consumption peaked and was attenuated by granisetron 24 h after cisplatin administration (day 7; P<0.05).

### Saccharin solution consumption in rats

**Figure 2:**
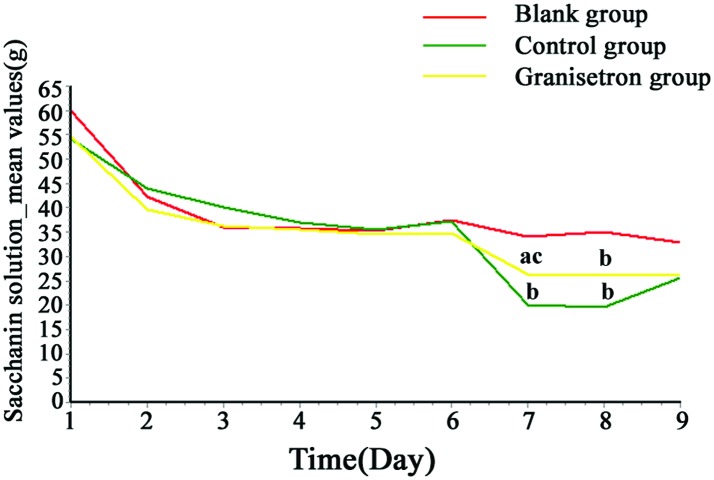
Effect of cisplatin administration on saccharin solution consumption in rats. Cisplatin significantly decreased saccharin solution consumption by days 7 and 8, and this effect was attenuated by granisetron administration (day 7). ^a^P<0.05, ^b^P<0.05 vs. blank group; ^c^P<0.05 vs. control group.

On day 1, saccharin solution consumption increased in all of the rats (~55–60 g/day), however, consumption returned to control level (~35–45 g/day) from day 2. During the adaptation period (days 1–6), there was no significant difference observed in saccharin solution consumption between the three groups (P>0.05). As demonstrated by Table II and Fig. 2, following the cisplatin injection, saccharin solution consumption in the control and granisetron groups decreased significantly when compared with the blank group at 24 and 48 h (day 7, P<0.05; day 8; P<0.01), although not at 72 h (day 9; P>0.05). Furthermore this decrease in saccharin solution consumption was attenuated by granisetron at 24 h (day 7; P<0.05), although not at 48 h (day 8; P>0.05).

### Normal feed consumption in rats

**Figure 3:**
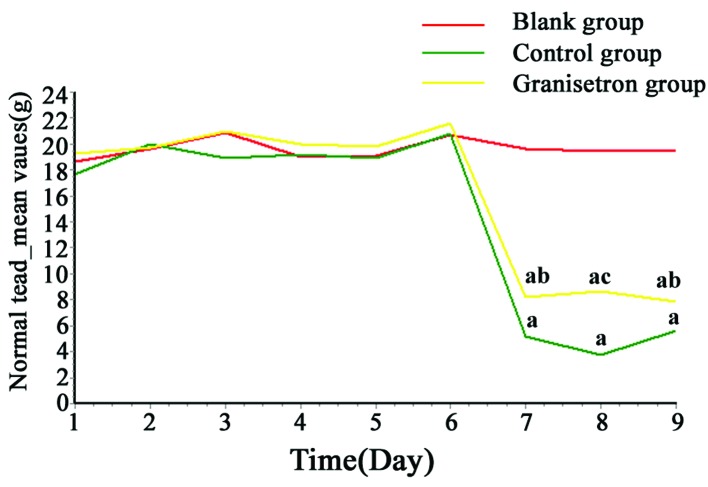
Effect of cisplatin administration on normal feed consumption in rats. Cisplatin significantly decreased normal feed consumption during the whole experimental period and this effect was attenuated by granisetron administration (days 7–9). ^a^P<0.01 vs. blank group; ^b^P<0.05, ^c^P<0.01 vs. control group.

Between days 1 and 6, there was no significant difference observed in normal feed consumption (P>0.05) between the three groups. As demonstrated by Table III and Fig. 3, following the cisplatin injection, normal feed consumption in the control and granisetron groups decreased significantly, when compared with the blank group at 24, 48 and 72 h (days 7–9; all P<0.01). Furthermore, this decrease was attenuated by granisetron administration during the whole experimental period (P<0.05).

## Discussion

Cisplatin is a chemotherapeutic agent, which is limited in its therapeutic administration by the unwanted side-effect of severe emesis. Cisplatin causes acute and delayed vomiting, classifying it into the highest emetic risk group, according to the American Society of Clinical Oncology guidelines (22). Acute vomiting predominantly occurs within a few minutes to several hours following cisplatin administration and commonly resolves within the first 24 h. Delayed vomiting develops in patients >24 h subsequent to cisplatin administration and persists for six to seven days (22). Furthermore, it is generally accepted that acute and delayed vomiting have different mechanisms. Acute vomiting is predominantly due to the release of serotonin within the gastrointestinal tract, whilst the delayed phase occurs following the release of substance P into the brainstem (23,24).

In contrast to species that possess an emetic reflex, rodents, such as rats exhibit pica in response to stimuli that induce emesis. Pica is an illness-response behavior, where consumption of non-nutritive substances, such as kaolin, is a phenomenon of the emetic reflex (25,26). Therefore, it has been proposed that pica may be analogous to emesis and kaolin consumption may be an index by which emesis may be evaluated in rats (25). It has been identified that various substances may induce an increase in kaolin consumption in rats, including oxycodone (27), apomorphine (4), cyclophosphamide, doxorubicin (28), cyclosporine A (29) and cisplatin (12,30,31). Previous studies in rats have found that in anticancer therapeutic agent-induced pica, the extent of kaolin intake and the duration of the altered feeding behavior, are associated with the clinical emetogenic potential of the therapeutic agent (32). Furthermore, intravenous or i.p. administration of cisplatin has been shown to induce a dose-dependent increase in kaolin consumption (28,33,34). However, the mechanism by which cisplatin induces pica is complicated and the duration of cisplatin-induced kaolin consumption remains controversial. It has been reported that administration of cisplatin at a dosage of 3 or 6 mg/kg significantly increased kaolin consumption within 0–24 h of the cisplatin injection, however, this significant increase did not extend beyond 24 h (8,19,35). Rudd *et al* (34), however, identified that the duration of kaolin consumption was associated with the dosage of cisplatin. For example, a low dose of cisplatin (3 mg/kg via i.p. injection) induced kaolin consumption during the 0–24 and 48–72 h periods, whilst the highest dose of cisplatin (6 mg/kg via i.p. injection) only induced kaolin consumption during the 0–24 h period. By contrast, considerable evidence indicates that cisplatin may induce a long-term (up to 48 h) increase in pica in rats (30,34,36–41), for example Saeki *et al* (33) reported that cisplatin-induced kaolin intake was observed for two days following cisplatin administration. Tatsushima *et al* (42), Mehendale *et al* (38) and Aung *et al* (43) demonstrated that cisplatin-induced kaolin intake could be induced for up to five days. De Jonghe *et al* (44) demonstrated that on days 1, 2, 4, 5 and 11 following a cisplatin injection, kaolin intake was significantly higher than in the comparable group, which was administered with saline; however, this increase significantly peaked on day 1 (~4 g/day kaolin) compared with the subsequent days (~1 g/day kaolin) (44). In the current study, it was identified that kaolin consumption was markedly enhanced one day after cisplatin administration (6 mg/kg), although not for more than one day.

The 5-HT_3_ receptor antagonist, ondansetron, has been identified as an inhibitor of cisplatin-induced pica in rodents (11,30). Saeki *et al* (33) reported that although kaolin intake was observed for two days following cisplatin administration, ondansetron only inhibited pica on the first day. In the current study, the duration of pica (following the cisplatin injection) did not exceed one day for the group administered with cisplatin (the control group), however, similar to ondansetron, granisetron inhibited cisplatin-induced pica on the first day only (the granisetron group). Together, these results indicate that kaolin consumption is the most appropriate method for evaluating acute (rather than delayed) emesis as an index of cisplatin-induced emesis, and that kaolin intake is only affected by the 5-HT_3_ receptor antagonist on day 1, which is consistent with the mechanism of acute emesis.

In animals, the combination of the taste and odor of the majority of foods provides a strong conditioned stimulus that can be utilised to elicit an appropriate response to any harmful unconditioned stimuli that may follow (45). CTA is, therefore, a behavioral response, which is essential to the survival of an individual. Drinkable sweet solutions, such as saccharin and sucrose, are the most widely used conditioned stimuli (46). Previously, CTA of rats was commonly used in studies of motion sickness (14,47).

Emetic agents, such as lithium chloride, have been shown to induce taste aversion in a diverse range of species (48,49), thus, lithium chloride has been used in hundreds of studies regarding CTA learning in rats (50,51). Previous investigations have also demonstrated that apomorphine, amphetamine and ethanol induce CTA in rats (52–54). There have, however, been few investigations of cisplatin-induced CTA in rats. Certain studies indicated that cisplatin was able to induce CTA in rats at doses that were known to induce emesis in other species; however, the responses were attenuated by dexamethasone and the rats were resistant to treatment with the 5-HT_3_ receptor antagonists (15,16), providing evidence that the 5-HT_3_ receptor antagonist is not involved in CTA mechanisms in rats (55). In the current study, considerable evidence indicated that rats were able to learn to avoid a taste, as saccharin solution consumption was greatly decreased two days subsequent to cisplatin administration. The current study did not observe an inhibitory effect of granisetron in CTA beyond 24 h, therefore, it was presumed that the 5-HT_3_ receptor antagonist was involved in the CTA mechanisms in the acute, but not in the delayed, emesis phase. Incidence of CTA in the delayed emesis phase may be due to other reasons, which are consistent with the mechanisms of delayed emesis. Furthermore, the duration of CTA indicates that saccharin solution consumption is effective in the evaluation of delayed emesis.

Anorexia nervosa is one of the most detrimental gastrointestinal side-effects associated with chemotherapy and is, therefore, used as an index of patient quality of life. A previous study showed that rats exhibit anorexic behavior in response to the administration of anticancer therapeutic agents and that the incidence of anorexia nervosa is not associated with the emetogenic potential of the rats (32). Therefore, the intake of normal feed was examined as an indicator of the emetic stimulus in rats (30,56,57). In cisplatin-treated rats, intracerebroventricular ghrelin administration (17), common hepatic branch vagotomy (1) and ondansetron (30) attenuated the decrease in food intake. In the current study, it was found that normal feed consumption decreased until at least the third day following cisplatin administration. The present study also indicated that reduced normal feed intake was attenuated by administration of the 5-HT_3_ receptor antagonist, granisetron during the whole experimental period. In conclusion, the results of the present study demonstrated that anorexia nervosa may be used to evaluate various phases of emesis, including the acute and delayed phases, and is a particularly important index of patient quality of life.

## Figures and Tables

**Table I:** Kaolin consumption induced by administration of cisplatin in rats.

		Kaolin consumption (g)		
		
Group	n	0–24 h	24–48 h	48–72 h
Blank	20	0.35±0.46	0.59±0.73	0.46±0.56
Control	20	2.45±1.46^a^	0.96±0.86	0.59±0.48
Granisetron	20	1.68±0.56^a^,^b^	0.72±0.55	0.30±0.18

a P<0.01 vs. blank group;

b P<0.05 vs. control group.

**Table II:** Saccharin solution consumption induced by administration of cisplatin in rats.

		Saccharin solution consumption (g)		
		
Group	n	0–24 h	24–48 h	48–72 h
Blank	20	34.18±9.99	34.95±11.41	32.78±9.65
Control	20	20.15±10.06^b^	19.57±11.47^b^	25.64±14.46
Granisetron	20	26.27±8.27^a^,^c^	26.03±7.27^b^	26.09±12.03

a P<0.05,

b P<0.01 vs. blank group;

c P<0.05 vs. control group.

**Table III:** Normal feed consumption induced by administration of cisplatin in rats.

		Normal feed consumption (g)		
		
Group	n	0–24 h	24–48 h	48–72 h
Blank	20	19.61±5.56	19.49±5.05	19.46±5.44
Control	20	5.19±2.15^a^	3.76±2.54^a^	5.63±2.18^a^
Granisetron	20	8.24±2.99^a^,^b^	8.61±3.18^a^,^c^	7.83±4.76^a^,^b^

a P<0.01 vs. blank group;

b P<0.05,

c P<0.01 vs. control group.

## References

[1] Rudd JA, Jordan CC, Naylor RJ (1996). The action of the NK1 tachykinin receptor antagonist, CP 99,994, in antagonizing the acute and delayed emesis induced by cisplatin in the ferret. Br J Pharmacol.

[2] Gardner CJ, Armour DR, Beattie DT (1996). GR205171: a novel antagonist with high affinity for the tachykinin NK1 receptor, and potent broad-spectrum anti-emetic activity. Regul Pept.

[3] Rudd JA, Naylor RJ (1996). An interaction of ondansetron and dexamethasone antagonizing cisplatin-induced acute and delayed emesis in the ferret. Br J Pharmacol.

[4] Takeda N, Hasegawa S, Morita M, Matsunaga T (1993). Pica in rats is analogous to emesis: an animal model in emesis research. Pharmacol Biochem Behav.

[5] De Jonghe BC, Horn CC (2008). Chemotherapy-induced pica and anorexia are reduced by common hepatic branch vagotomy in the rat. Am J Physiol Regul Integr Comp Physiol.

[6] Garcia J, Hankins WG, Rusiniak KW (1974). Behavioral regulation of the milieu interne in man and rat. Science.

[7] Liu Y, Hamaue N, Endo T, Hirafuji M, Minami M (2003). 5-hydroxytryptamine (5-HT) concentrations in the hippocampus, the hypothalamus and the medulla oblongata related to cisplatin-induced pica of rats. Res Commun Mol Pathol Pharmacol.

[8] Horn CC, De Jonghe BC, Matyas K, Norgren R (2009). Chemotherapy-induced kaolin intake is increased by lesion of the lateral parabrachial nucleus of the rat. Am J Physiol Regul Integr Comp Physiol.

[9] Saito R, Takano Y (2006). Easy method for emesis using rats. Nihon Yakurigaku Zasshi.

[10] Mehendale SR, Aung HH, Yin JJ (2004). Effects of antioxidant herbs on chemotherapy-induced nausea and vomiting in a rat-pica model. Am J Chin Med.

[11] Yamamoto K, Matsunaga S, Matsui M, Takeda N, Yamatodani A (2002). Pica in mice as a new model for the study of emesis. Methods Find Exp Clin Pharmacol.

[12] Takeda N, Hasegawa S, Morita M (1995). Neuropharmacological mechanisms of emesis. II. Effects of antiemetic drugs on cisplatin-induced pica in rats. Methods Find Exp Clin Pharmacol.

[13] Liu YL, Malik N, Sanger GJ, Friedman MI, Andrews PL (2005). Pica - a model of nausea? Species differences in response to cisplatin. Physiol Behav.

[14] Cai YL, Ma WL, Li M (2005). Behavioral changes of rats under rotation stimulation. Space Med Med Eng (Beijing).

[15] Rudd JA, Ngan MP, Wai MK (1998). 5-HT3 receptors are not involved in conditioned taste aversions induced by 5-hydroxytryptamine, ipecacuanha or cisplatin. Eur J Pharmacol.

[16] Mele PC, McDonough JR, McLean DB, O’Halloran KP (1992). Cisplatin-induced conditioned taste aversion: attenuation by dexamethasone but not zacopride or GR38032F. Eur J Pharmacol.

[17] Yakabi K, Sadakane C, Noguchi M (2010). Reduced ghrelin secretion in the hypothalamus of rats due to cisplatin-induced anorexia. Endocrinology.

[18] Ando K, Takagi K, Tsubone H (2012). Enhanced gastric retention of solid resin beads as a marker for emetic potential of agents in rats. J Toxicol Sci.

[19] Cabezos PA, Vera G, Castillo M (2008). Radiological study of gastrointestinal motor activity after acute cisplatin in the rat. Temporal relationship with pica. Auton Neurosci.

[20] Mitchell D, Wells C, Hoch N (1976). Poison induced pica in rats. Physiol Behav.

[21] Aung HH, Mehendale SR, Xie JT, Moss J, Yuan CS (2004). Methylnaltrexone prevents morphine-induced kaolin intake in the rat. Life Sci.

[22] Kris MG, Hesketh PJ, Somerfield MR, American Society of Clinical Oncology (2006). American Society of Clinical Oncology guideline for antiemetics in oncology: update 2006. J Clin Oncol.

[23] Rojas C, Li Y, Zhang J (2010). The antiemetic 5-HT3 receptor antagonist Palonosetron inhibits substance P-mediated responses in vitro and in vivo. J Pharmacol Exp Ther.

[24] Darmani NA, Crim JL, Janoyan JJ, Abad J, Ramirez J (2009). A re-evaluation of the neurotransmitter basis of chemotherapy-induced immediate and delayed vomiting: evidence from the least shrew. Brain Res.

[25] Yamamoto K, Ngan MP, Takeda N, Yamatodani A, Rudd JA (2004). Differential activity of drugs to induce emesis and pica behavior in *Suncus murinus* (house musk shrew) and rats. Physiol Behav.

[26] Vera G, Chiarlone A, Cabezos PA (2007). WIN 55,212-2 prevents mechanical allodynia but not alterations in feeding behaviour induced by chronic cisplatin in the rat. Life Sci.

[27] Batra VR, Schrott LM (2011). Acute oxycodone induces the pro-emetic pica response in rats. J Pharmacol Exp Ther.

[28] Jeong SW, Cho JW, Hwang JS (2005). The antiemetic effect of a novel tropisetron patch in anticancer agents-induced kaolin pica model using rats. Environ Toxicol Pharmacol.

[29] Fujisaki Y, Yamauchi A, Shuto H (2001). Pharmacological characterization of cyclosporine A-induced kaolin intake in rats. Pharmacol Biochem Behav.

[30] Malik NM, Liu YL, Cole N, Sanger GJ, Andrews PL (2007). Differential effects of dexamethasone, ondansetron and a tachykinin NK1 receptor antagonist (GR205171) on cisplatin-induced changes in behaviour, food intake, pica and gastric function in rats. Eur J Pharmacol.

[31] Vera G, Chiarlone A, Martín MI, Abalo R (2006). Altered feeding behaviour induced by long-term cisplatin in rats. Auton Neurosci.

[32] Yamamoto K, Nakai M, Nohara K, Yamatodani A (2007). The anti-cancer drug-induced pica in rats is related to their clinical emetogenic potential. Eur J Pharmacol.

[33] Saeki M, Sakai M, Saito R (2001). Effects of HSP-117, a novel tachykinin NK1-receptor antagonist, on cisplatin-induced pica as a new evaluation of delayed emesis in rats. Jpn J Pharmacol.

[34] Rudd JA, Yamamoto K, Yamatodani A, Takeda N (2002). Differential action of ondansetron and dexamethasone to modify cisplatin-induced acute and delayed kaolin consumption (‘pica’) in rats. Eur J Pharmacol.

[35] Cabezos PA, Vera G, Martín-Fontelles MI, Fernández-Pujol R, Abalo R (2010). Cisplatin-induced gastrointestinal dysmotility is aggravated after chronic administration in the rat. Comparison with pica. Neurogastroenterol Motil.

[36] Horn CC, Ciucci M, Chaudhury A (2007). Brain Fos expression during 48 h after cisplatin treatment: neural pathways for acute and delayed visceral sickness. Auton Neurosci.

[37] Wang CZ, Basila D, Aung HH (2005). Effects of *Ganoderma lucidum* extract on chemotherapy-induced nausea and vomiting in a rat model. Am J Chin Med.

[38] Mehendale S, Aung H, Wang A (2005). American ginseng berry extract and ginsenoside Re attenuate cisplatin-induced kaolin intake in rats. Cancer Chemother Pharmacol.

[39] Du J, Li P, Sun X, Zhang M (2011). Ameliorative effect of *Armillariella tabescens* on cisplatin-induced gastrointestinal tract reaction in the rat. Zhonghua Zhong Liu Za Zhi.

[40] Qian Q, Chen W, Guo C (2011). Xiao-Ban-Xia-Tang inhibits cisplatin-induced pica by down regulating obestatin in rats. J Ethnopharmacol.

[41] Wang CZ, Fishbein A, Aung HH (2005). Polyphenol contents in grape-seed extracts correlate with antipica effects in cisplatin-treated rats. J Altern Complement Med.

[42] Tatsushima Y, Egashira N, Matsushita N (2011). Pemirolast reduces cisplatin-induced kaolin intake in rats. Eur J Pharmacol.

[43] Aung HH, Dey L, Mehendale S (2003). *Scutellaria baicalensis* extract decreases cisplatin-induced pica in rats. Cancer Chemother Pharmacol.

[44] De Jonghe BC, Lawler MP, Horn CC, Tordoff MG (2009). Pica as an adaptive response: Kaolin consumption helps rats recover from chemotherapy-induced illness. Physiol Behav.

[45] Cairnie AB, Leach KE (1982). Dexamethasone: a potent blocker for radiation-induced taste aversion in rats. Pharmacol Biochem Behav.

[46] Geerse GJ, van Gurp LC, van Wijk DC, Wiegant VM, Stam R (2007). Duodenal pain and spinal morphine induce conditioned taste aversion in rats. Physiol Behav.

[47] Wu XW, Xin B, Zou JF (2012). Effect of rotation stimulation on the anesthetic sensitivity of sevoflurane in rats. Zhongguo Ying Yong Sheng Li Xue Za Zhi.

[48] Garcia J, Rusiniak KW, Brett LP, Davis H, Hurwitz H (1977). Conditioning food-illness aversions in wild animals: Caveant canonici. Operant-Pavlovian Interactions.

[49] Smith JE, Friedman MI, Andrews PL (2001). Conditioned food aversion in *Suncus murinus* (house musk shrew) - a new model for the study of nause in a species with an emetic reflex. Physiol Behav.

[50] Cross-Mellor SK, Ossenkopp KP, Piomelli D, Parker LA (2007). Effects of the FAAH inhibitor, URB597, and anandamide on lithium-induced taste reactivity responses: a measure of nausea in the rat. Psychopharmacology (Berl).

[51] Riley AL, Freeman KB (2004). Conditioned taste aversion: a database. Pharmacol Biochem Behav.

[52] McAllister KH, Pratt JA (1998). GR205171 blocks apomorphine and amphetamine-induced conditioned taste aversions. Eur J Pharmacol.

[53] Anderson RI, Agoglia AE, Morales M, Varlinskaya EI, Spear LP (2013). Stress, κ manipulations, and aversive effects of ethanol in adolescent and adult male rats. Neuroscience.

[54] Shinpo K, Hirai Y, Maezawa H, Totsuka Y, Funahashi M (2012). The role of area postrema neurons expressing H-channels in the induction mechanism of nausea and vomiting. Physiol Behav.

[55] Bienkowski P, Kuca P, Piasecki J, Kostowski W (1997). 5-HT3 receptor antagonist, tropisetron, does not influence ethanol-induced conditioned taste aversion and conditioned place aversion. Alcohol.

[56] Raghavendran HR, Rekha S, Shin JW (2011). Effects of Korean ginseng root extract on cisplatin-induced emesis in a rat-pica model. Food Chem Toxicol.

[57] Liu YL, Malik NM, Sanger GJ, Andrews PL (2006). Ghrelin alleviates cancer chemotherapy-associated dyspepsia in rodents. Cancer Chemother Pharmacol.

